# RNA velocity—current challenges and future perspectives

**DOI:** 10.15252/msb.202110282

**Published:** 2021-08-26

**Authors:** Volker Bergen, Ruslan A Soldatov, Peter V Kharchenko, Fabian J Theis

**Affiliations:** ^1^ Institute of Computational Biology Helmholtz Center Munich Munich Germany; ^2^ Department of Mathematics Technical University of Munich Munich Germany; ^3^ Department of Biomedical Informatics Harvard Medical School Boston MA USA

**Keywords:** challenges, dynamics, limitations, perspectives, RNA velocity, Chromatin, Epigenetics, Genomics & Functional Genomics, Computational Biology

## Abstract

RNA velocity has enabled the recovery of directed dynamic information from single‐cell transcriptomics by connecting measurements to the underlying kinetics of gene expression. This approach has opened up new ways of studying cellular dynamics. Here, we review the current state of RNA velocity modeling approaches, discuss various examples illustrating limitations and potential pitfalls, and provide guidance on how the ensuing challenges may be addressed. We then outline future directions on how to generalize the concept of RNA velocity to a wider variety of biological systems and modalities.

## Graphical abstract

This Review discusses the emerging challenges and potential pitfalls of current RNA velocity modeling approaches and provides guidance on how to address them.

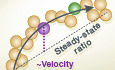

## Background

**Figure 1:**
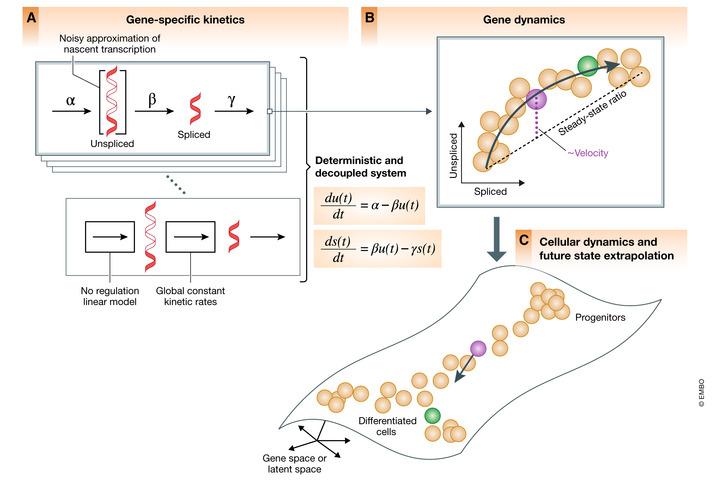
Current state of RNA velocity modeling (A) Transcription of pre‐mRNAs, their conversion into spliced mRNAs, and eventual degradation. Current RNA velocity modeling approaches use basic reaction kinetics for each gene independently and formulate deterministic differential equations with linear dependencies, assuming constant rates. The system is decoupled across genes and does not account for transcriptional regulation. (B) The temporal response delay of pre‐mRNA being spliced into mature mRNA manifests itself in the curvature in phase space and is leveraged to model and estimate RNA velocity for each gene. Velocity is obtained from the residual of the observed ratio to the inferred steady‐state ratio, i.e., the ratio of degradation to splicing rate. (C) The combination of velocities across genes is used to extrapolate the future state of an individual cell.

A central challenge in studying cellular dynamics in single‐cell genomics is that single‐cell RNA‐seq provides only static snapshots of cellular states at the moment of the measurement, instead of following cells over time. The concept of RNA velocity (La Manno *et al*, 2018) has unlocked new ways of studying cellular dynamics by granting access to not only the descriptive state of a cell, but also to its direction and speed of movement in transcriptome space, thereby enabling predictive models of cell dynamics. RNA velocity recovers directed information by distinguishing newly transcribed pre‐mRNAs (unspliced) from mature mRNAs (spliced), which can be detected in standard single‐cell RNA‐seq protocols from the presence of introns. The change in mRNA abundance, termed RNA velocity, is inferred by a per‐gene reaction model that relates the abundance of unspliced and spliced mRNA (Fig 1A). Positive velocity indicates a recent increase in unspliced transcripts (thus abundances being higher than expected in steady state) followed by up‐regulation in spliced transcripts. Conversely, negative velocity indicates down‐regulation (Fig 1B). The combination of velocities across genes is then used to estimate the future state of an individual cell (Fig 1C).

Recent advances have extended the concept to dynamic populations and enabled inference of reaction rates, reconstruction of time, and detection of transiently expressed genes from the underlying kinetics (Bergen *et al*, 2020). It has been shown that a small subset of dynamical genes commonly informs the reconstruction of the entire velocity vector field. This observation illustrated that in most scenarios, only a small number of genes appear to obey simple interpretable kinetics used by RNA velocity, which creates a major challenge in interpreting RNA velocity results. While RNA velocity has been taken up in a series of applications as summarized recently (Lederer & La Manno, 2020); here, we focus on its underlying modeling concepts, limitations, and possible extensions. In particular, we discuss issues that can lead to misspecification of transcriptional models and outline potential conceptual and technical model extensions that may resolve these limitations and generalize the concept of RNA velocity. Our documented case study can be found at: https://scvelo.org/perspectives.

## Current state, model assumptions, and potential pitfalls

Currently, two modeling approaches exist that leverage expression kinetics to estimate RNA velocity—the originally proposed “steady‐state” model *velocyto* and the subsequently extended dynamical model *scVelo*. The steady‐state model (La Manno *et al*, 2018) estimates velocities as the deviation of the observed ratio of unspliced to spliced mRNA from an inferred steady‐state ratio. The steady‐state ratio is approximated with a linear regression on cells found in the lower and upper quantiles where they are expected to have reached steady‐state expression levels. This model makes two central assumptions: a common splicing rate across genes and the presence of at least partial observation of the steady‐state expression levels in the sampled data. Although providing robust estimation, these assumptions may lead to errors in velocity estimates and cellular states when they are violated, e.g., due to heterogeneous subpopulation or inability to observe the system near its steady state. The likelihood‐based dynamical model, introduced recently, generalizes velocity estimation to transient systems (Bergen *et al*, 2020). While it relaxes the steady‐state assumption, it remains that the kinetics are explained with a deterministic and fully decoupled system of linear differential equations with constant kinetic rate parameters. Beyond the scope of computational modeling, the statistical power of the methods depends on the curvature in the phase portrait since a lack of curvature challenges current models to distinguish whether an up‐ or down‐regulation is occurring. The overall curvature of deviation from the steady‐state line in the phase portrait is mostly impacted by the ratios of splicing to degradation rates (Box 1), indicating that statistical inference is limited to genes where splicing is faster or comparable to degradation, while a small ratio would yield straight lines rather than an interpretable curvature. Note, that this lack of signal is highly gene‐specific. Another source of ambiguity only revealing straight lines is the incomplete scope of observation of dynamic processes, which we frequently find in subpopulations because of partially observed expression kinetics, e.g., being upregulated only at the very end or downregulated at the very beginning of a process.

**Figure 2:**
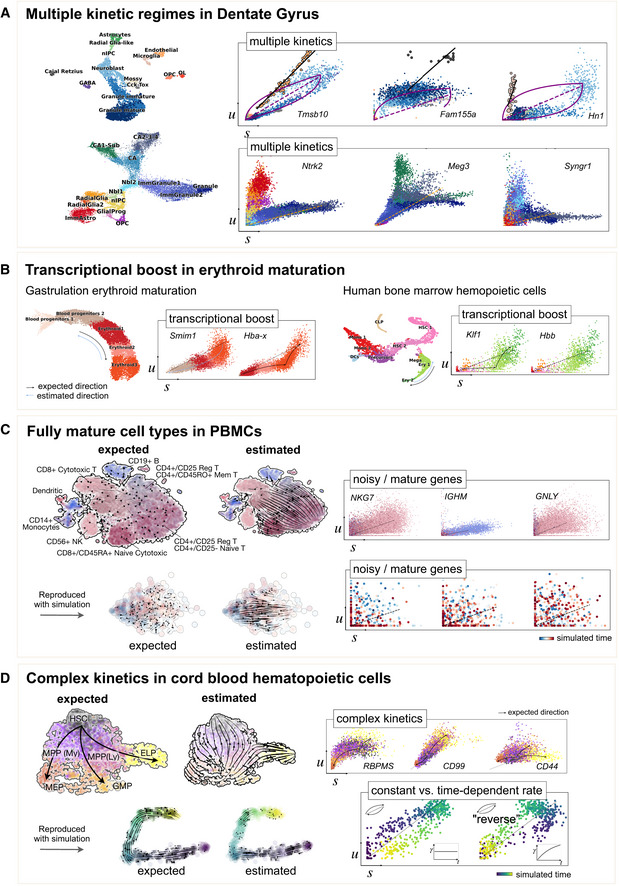
Examples illustrating limitations of current RNA velocity models (A) A UMAP‐based representation (left) and gene unspliced/spliced phase portraits (right) of Dentate Gyrus neurogenesis, adapted and reanalyzed from Bergen *et al*, 2020 (Suppl. Fig 11) and La Manno *et al*, 2018 (Suppl. Fig 7). These genes show multiple kinetic regimes across subpopulations and lineages, possibly governed by different kinetic rates, and manifested as multiple trajectories/slopes. For instance, the endothelial subpopulation in *Tmsb10* yields positive velocity estimates indicating up‐regulation, although it can be unambiguously estimated given only a slope distinct from the main granule lineage. To resolve these multiple regimes, it requires a model that identifies these regimes and allows for variable kinetic rates. (B) Erythroid maturation in mouse gastrulation (top) and human bone marrow CD34^+^ hematopoietic cells (bottom) that show transcriptional boosts in expression possibly induced by a change in transcription rate. Data from Setty *et al* (2019), Barile *et al* (2021). (C) Peripheral blood mononuclear cells (PBMCs) from Zheng et al (2017) with mature cell types. Arbitrary directions are projected onto the UMAP representation (left) even though velocity estimates are used from three genes only (right) that show no transient states. Expected would have been a noisy vector field that is not pointing into any particular direction. That shows the possibility of false projections that are not supported by gene‐wise dynamics. Simulated data of mature cell types support this observation of possible false projections that are not seen in the ground‐truth vector field. (D) Cord blood CD34^+^ hematopoietic cells with complex kinetics that shows simultaneous up‐ and down‐regulation during the transition from HSCs toward different fates of megakaryocyte/erythrocyte (MEPs), granulocyte/macrophage (GMPs), and early lymphocyte progenitors (ELP). *RBPMS* even shows misleading concavity patterns causing a direction reversal. The possibility of reserved directions can be explained by time‐dependent degradation rates, as demonstrated using simulated data. CD34^+^ cord blood cell data are unpublished.

**Figure 3:**
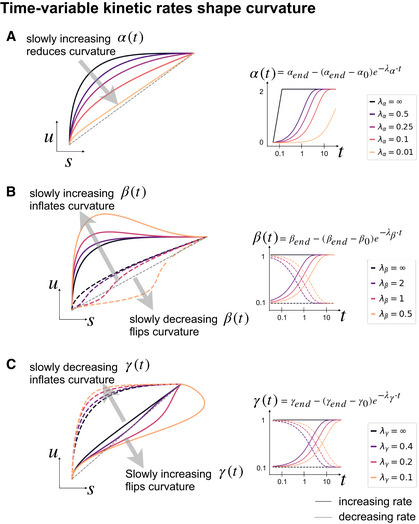
Time‐variable kinetic rates shape curvature of gene activation (A) Time‐dependent kinetic rates shape the curvature patterns of gene activation. A slow increase in transcription rate rather than a stepwise activation deflates the curvature and thus decreases the statistical power. (B) A slow increase in splicing rates inflates the curvature while a slow decrease in splicing rates flips the curvature. That results in a convex curve, which yields negative velocities and gets incorrectly interpreted as down‐regulation. In the worst case, this can also cause a direction reversal in the projected velocities. (C) The impact of time‐dependent degradation rates is inverse to time‐dependent splicing rates. A slow decrease in degradation rates inflates the curvature while a slow increase in degradation rates flips the curvature.

To demonstrate potential pitfalls, we provide several examples that disclose different types of limitations of current modeling approaches (Fig 2). First, as described in the seminal works (La Manno *et al*, 2018; Bergen *et al*, 2020), some genes show multiple kinetic regimes across subpopulations and lineages (Fig 2A). These can be governed by variations in splicing to degradation rates ratios and manifest as multiple trajectories in phase space. Second, as recently shown in mouse gastrulation (Pijuan‐Sala *et al*, 2019; Barile *et al*, 2021), a boost in expression has been observed in erythroid maturation, possibly induced by a change in transcription rate (Fig 2B). We made the same observation in human bone marrow CD34^+^ hematopoietic cells (Setty *et al*, 2019). This up‐regulating boost in expression would incorrectly yield negative velocity estimates indicating down‐regulation. Third, a common example of incomplete scope is the observation of only steady‐state populations. Thus, we examined erroneously inferred directions in terminal cell types in PBMCs (Zheng *et al*, 2017), where we would not have expected any explicit cell type transition (Fig 2C). Genes not showing any transient states can be explained by high noise levels. However, in this example it is more likely that cells are mostly sampled in mature states, where mRNA levels have already equilibrated and intermediate states leading to these equilibria have not been sampled. Despite the lack of dynamic information, we still obtain arbitrary erroneous directions. To confirm that these directions indeed arise from distorted estimates and their projection, we show that the directions were also inferred even if using three top‐likelihood selected genes only (*NKG7*, *IGHM*, and *GNLY*) all of which show noisy phase portraits without any indication of cell type transitions. Hence, the unexpected projected directions are likely due to velocities being estimated independently of noise levels and uncertainty in estimates not being propagated into the projection. A simulation of mature cell types further supports the possibility of false projections as projected arrows are obtained that are not seen in the ground‐truth vector field (Fig 2C). Finally, we investigated potential issues in hematopoiesis, using cord blood CD34^+^ cells, where we obtain a direction reversal from what is biologically expected. In *CD99* and *CD44*, we observe complex characteristics that cannot be resolved by current models: a simultaneous up‐ and down‐regulation during their transition from HSCs toward different fates. In *RBPMS*, we find misleading concavity patterns where we would have expected a convex curve, causing a direction reversal not only gene‐specific but even in the projected arrows (Fig 2D), which can be explained by time‐dependent rates. Experimental approaches that started elucidating time‐dependent mRNA turnover reveal frequent modulation of kinetic rates in time (Battich *et al*, 2020). Motivated by these examples, we explored how time‐dependent kinetic rates shape the curvature of gene activation. Simulations show how time‐dependent rates can reshape curvature patterns: Variable synthesis rates deflate curvature (Fig 3A); slowly decreasing degradation and increasing splicing rates inflate curvature, while slowly increasing degradation and decreasing splicing rates flip curvature (Fig 3B and C).

**Box 1: Kinetic signal (overall curvature) is determined by the ratio of splicing and degradation rate, and the rate of transcription convergence**

Consider the differential equation

$$\frac{du}{dt}=\alpha ‐\beta u,\frac{ds}{dt}=\delta u‐\gamma s,$$

where the splicing rate parameters β and δ are treated differently for generality to account for technical effects such as amplification biases.

The analytical solution is given by

$$u\left(t\right)=u_{0}e^{‐\gamma t}+\frac{\alpha }{\beta }\left(1‐e^{‐\beta t}\right),$$

$$s\left(t\right)=s_{0}e^{‐\gamma t}+\frac{\delta }{\beta }\frac{\alpha }{\gamma }\left(1‐e^{‐\gamma t}\right)+\frac{\delta }{\beta }\frac{\alpha ‐\beta u_{0}}{\gamma ‐\beta }\left(e^{‐\gamma t}‐e^{‐\beta t}\right).$$

The kinetic signal is given by the concavity of the residuals (for up‐regulation, while convexity for down‐regulation). Assuming $s_{0}=u_{0}=0$, the residuals are given by

$$r\left(t\right)=\left|u‐\frac{\gamma }{\delta }s\right|=\frac{\alpha }{\gamma ‐\beta }\left(e^{‐\beta t}‐e^{‐\gamma t}\right).$$

The overall deviation from the equilibrium line is given by integration over the residuals

$$C=\int_{0}^{\infty }r\left(t\right)ds\left(t\right)=\int_{0}^{\infty }r\left(t\right)\frac{ds}{dt}dt=\frac{1}{2}\frac{\beta }{\gamma +\beta }\frac{\alpha }{\beta }\frac{\delta \alpha }{\beta \gamma }=\frac{1}{2}\frac{\beta }{\gamma +\beta }s_{\mathrm{steady}}u_{\mathrm{steady}}.$$

When allowing a time‐dependent gradually increasing transcription rate $\alpha \left(t\right)=\alpha \left(1‐e^{‐\lambda_{\alpha }t}\right),$ then the overall curvature is given by

$$C\left(\lambda_{\alpha }\right)=C\cdot \left(1‐\frac{\beta \gamma }{\left(\beta +\lambda_{\alpha }\right)\left(\gamma +\lambda_{\alpha }\right)}\right).$$

These equations have three important implications:

1. $\frac{\beta }{\gamma +\beta }$ is the kinetic characteristic of statistical power, which notably depends only on the unbiased rate parameters of splicing and degradation, ranging from 0 (straight line) to 1 (maximally pronounced curvature).
2. $s_{\mathrm{steady}}u_{\mathrm{steady}}$ is the detection power, which is important for practical settings as noise levels can be regarded as a function of expression levels.
3. A gradual increase in synthesis rate through $\lambda_{\alpha }$ deflates the curvature pattern.

## Conceptual extensions and future directions

**Figure 4:**
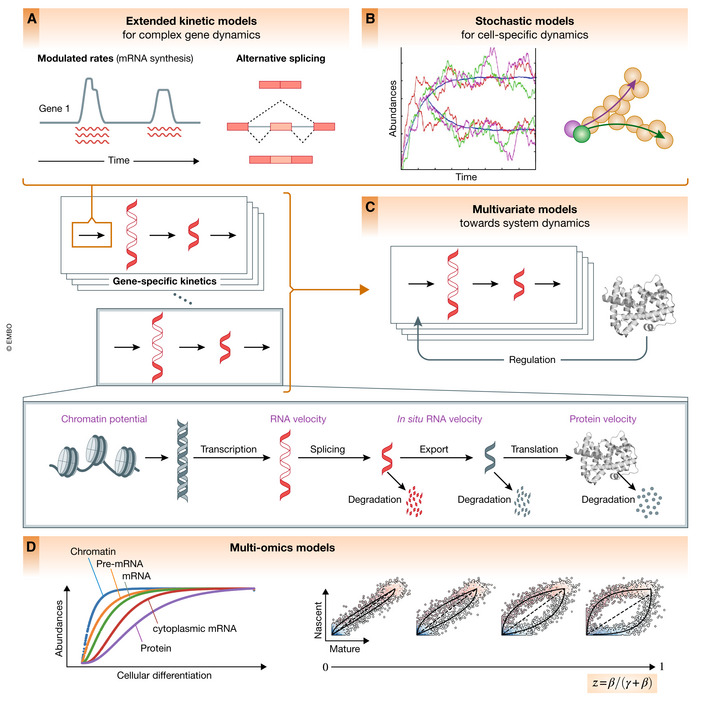
Conceptual future directions and model extensions (A) Modulations of transcription, splicing, and degradation rates by more complex mechanisms, including transcriptional bursts, alternative splicing events, and regulation of mRNA stability, suggest extended kinetic extensions such as modeling time‐ and state‐variable kinetic rates. (B) Stochastic variability may be leveraged to capture the bursting nature of transcription, to improve parameter identifiability and to identify other sources of heterogeneity in kinetic rates that can be informative during cell fate decisions, when epigenetic priming or environmental signals guide cellular decisions. (C) The gene expression model can be extended to not only describe cell‐state transitions, but also regulatory interactions along these transitions. (D) In addition to exonic and intronic signals, other molecular moieties can be incorporated into the model, such as protein measurements, metabolically labeled mRNAs, cytoplasmic mRNA, or chromatin state. The statistical signal as defined by the curvature is mostly determined by the ratio of rates at which the expression levels of the two modalities decay (ratio of splicing and degradation for RNA velocity), which may be improved through incorporation of other moieties.

Most of the challenges can be addressed with conceptual model extensions. Here, we will describe possible extensions to account for more complex kinetics, stochasticity, gene regulation, multivariate, and omics readouts (Fig 4).

### Extended kinetic models for gene dynamics

The transcriptional kinetics is currently modeled as simple first‐order equations with constant kinetic rate parameters. The fact that only a subset of genes follows simple kinetics is partly due to modulations in transcription, splicing, and degradation rates by more complex mechanisms. Kinetic rates can be dynamically regulated as demonstrated in neurogenesis and hematopoiesis (Fig 2A and B). In particular, recent metabolic RNA‐labeling experiments, which quantify preexisting and labeled newly synthesized transcripts at a single‐cell level, uncovered diverse behaviors of kinetic rates during in vitro differentiation of intestinal stem cells and cell cycle (Battich *et al*, 2015). Variable kinetic rates either between cell states or during a dynamic process can lead to phase portraits that have a misleading interpretation through the lens of existing RNA velocity models. We expect extensions of RNA velocity kinetic modeling that account for dynamic changes in kinetic rates (Fig 4A). These models will improve the quality of RNA velocity predictions, when accounting for alternative processes that modulate the transcription machinery, splicing, and mRNA stability. Additionally, such state‐variable models will provide insights into transcriptional and post‐transcriptional regulatory processes that control gene expression dynamics. The latter may also enable kinetics to be modeled in time series designs. If the underlying kinetic rate parameters are state‐dependent, thus discretely changing, it should be possible to identify them upon classifying cells into their kinetic regimes. Identification of time‐variable rates, however, will require additional constraints such as a pseudotime prior, optimal transport with marginal constraints in time course measurements (Schiebinger *et al*, 2019), or some other form of regularization. Finally, statistical quantification of changes in kinetic rates of analogous cell types under different conditions (e.g., health vs. disease) will allow us to identify condition‐specific dynamics.

### Stochastic models for cell‐specific dynamics

Expression kinetics are inherently stochastic, driven by random biophysical interactions involved in the activity of the RNA synthesis and turnover machinery. The randomness of such biomolecular interactions coupled with the seemingly contradictory aspect of precise coordination allows cells to explore broader regimes, e.g., to differentiate toward multiple fates. Such mechanisms include the bursting nature of transcription, which indicates stochastic synthesis rates. Similarly, the noise induced by small copy numbers of a given transcript in a cell and the limited amount of material available per cell contribute to variations across cells and, consequently, variations in cellular decision making. While in systems biology, it has been shown that these may be leveraged for better model identification (Munsky *et al*, 2009) or in the modeling of cellular decision making using diffusion processes (Haghverdi *et al*, 2016), this stochasticity is currently ignored in RNA velocity modeling: The models describe the kinetics by deterministic differential equations, which do not allow to identify other sources of heterogeneity in kinetic rates, such as those imposed by external factors or unmeasured internal cell properties (Hahl & Kremling, 2016). These sources of heterogeneity can have important implications and may be informative during cell fate decisions (Raj & van Oudenaarden, 2008), when epigenetic priming or environmental signals guide different cellular decisions of transcriptionally similar cells, e.g., at decision forks (Soldatov *et al*, 2019). While RNA velocity provides a local estimate of cellular kinetics, global cell fate trajectories may be inferred through Markov chain transitions along the expression manifold (La Manno *et al*, 2018; Bergen *et al*, 2020) or between cellular states (preprint: Lange *et al*, 2020), which we expect to further improve when explored at the level of stochastic kinetic modeling. In the future, we are expecting non‐deterministic models of RNA velocity, thus allowing improved detection rates to account for cell type‐specific or even cell‐specific kinetic rates (Fig 4B). The resulting more accurate single‐cell estimates will further enable us to move from a deterministic limit to an estimated distribution of possible directions of a cell in an observed state, e.g., to facilitate cell fate bifurcation analysis. Such stochastic, cell‐specific models, combined with the inference of cell division and death rates, will further enable dynamic inference over large expression manifolds and a better understanding of transitions between cellular states.

### Multivariate models toward system dynamics

Dynamic changes in gene expression are orchestrated by transcriptional and post‐transcriptional regulations. As shown in the example of erythroid maturation from gastrulation and human bone marrow, a transcriptional boost in expression can be induced by some upstream regulators (Fig 2B). At the current stage, the model for transcriptional dynamics is fully decoupled; i.e., each gene is treated independently, and regulatory relationships are ignored. The dynamical gene expression model can be extended to a multivariate model that describes not only cell‐state transitions, but also regulatory interactions along these transitions. Regulatory events can be observed statistically in expression changes along pseudotime. To describe these events, the expression patterns of target genes can be modeled as a function of transcription factor activities, ideally treated as a nonlinear system, for instance, using Hill kinetics. A comprehensive evaluation of network modeling algorithms demonstrates that none of the currently available methods are capable of accurately recovering network structures from single‐cell expression data alone, and the effort of inferring gene regulatory networks is still in its infancy (Pratapa *et al*, 2020). A recent analysis, however, indicates that the inclusion of RNA velocity information enables at least partial recovery of a regulatory network compared with pseudotime‐based approaches (Qiu *et al*, 2020). It opens an avenue to generative approaches that model the known mRNA velocities as a function of expression state to infer the underlying gene regulatory network (Fig 4C). Using learned networks, we can generate new trajectories and testable hypotheses from transcription factor activity, for instance, to understand perturbational responses. Finally, an ultimate multivariate approach would jointly model the unknown RNA velocities and the underlying regulatory network from observed expression states and interpretable models of expression kinetics. Although efficient inference of the coupled system may quickly become challenging, such a joint model allows us to better understand fate decisions and reveal regulatory mechanisms of lineage priming. Furthermore, technological advances and the inclusion of new functional genomic layers, such as transcription factor binding, regulatory sequence motifs, chromatin modifications, and intermediaries such as RNA polymerase activity, hold great promise. These additional readouts will provide informative priors on the regulatory network and extend specifications of kinetic models.

### Multi‐modal omics models

RNA velocity is grounded in connecting measurements to an underlying mechanism (mRNA splicing), with two modalities representing the current and future state. In addition to exonic and intronic signals, other omics and molecular moieties can be leveraged if such measurements are available in an unbiased manner (Lederer & La Manno, 2020). Exploring other modalities becomes particularly crucial for systems, where the transcriptional dynamics of mRNA splicing does not provide sufficient signal, e.g., if splicing rate is relatively small as opposed to a large degradation rate (Box 1, Fig 4D). This issue of insufficient signal presents a challenge for the current mRNA splicing models, but may be resolvable, for instance, through analysis of other modalities, e.g., using protein dynamics, where we could expect the kinetic characteristic of statistical power (Box 1) to increase from 0.5 to 0.8 (Fig 4D), when assuming a fivefold half‐life in proteins as opposed to RNA. For moieties such as capped, polyadenylated, and degraded transcript fragments or protein abundance, the model extension is straightforward upon revising the underlying assumptions and moiety‐specific statistical model while ensuring reliable quantification. Experimental information on the molecular compartments such as separation of nuclear vs. cytoplasmic balance (Xia *et al*, 2019) using spatially resolved MERFISH protocol can also be incorporated into the model. Furthermore, models can be extended to incorporate epigenetic and regulatory information based on single‐cell chromatin accessibility or other epigenetic data (Ma *et al*, 2020).

Ultimately, velocity estimation relies on accurate quantification of abundances. Experiments indicate that intronic reads are only noisy approximations of nascent transcription (Erhard *et al*, 2019) and approaches for improving this quantification would be helpful. On the experimental side, relative abundances can be directly inferred using *in vitro* metabolic labeling (Erhard *et al*, 2019; Battich *et al*, 2020; Cao *et al*, 2020). This additional readout can be included in the dynamical model, incorporating varying labeling lengths as additional priors. It may also be possible to boost the detection of intronic molecules or reduce background from non‐coding and antisense RNAs through improved preprocessing steps. On the computational side, additional structural features of the reads and gene‐specific models of spliced vs. unspliced read patterns may improve the signal‐to‐noise ratio (Fig 4D).

## Technical challenges and extensions

Here, we outline technical challenges that impact the modeling, such as normalization, batch effects, and gene selection, and in parts discuss how to address them.

### Cell size normalization

Current RNA velocity approaches provide normalization by size factors proportional to the count depth per cell, and variations of such. However, cell size also reflects the natural extension of the reservoir of RNA transcription. It is not entirely clear how to best account for the cell count depth, whether to normalize intronic and exonic matrices to matrix‐specific factors, to shared factors, or even to not normalize at all. More generally, we should investigate how changes in global cellular parameters, such as splicing efficiency or abundance of RNA polymerases, affect the kinetic models. Normalization by cell size is a simple way to remove the effects of count sampling, but it can also distort these effects in a non‐trivial manner. Adequate preprocessing and ideally the inclusion of these effects into the model are crucial for accurate velocity estimates.

### Estimation from single‐nucleus data

Transcriptional measurements from individual nuclei enable the analysis of tissues where whole cell isolation is challenging (Slyper *et al*, 2020). The physical isolation of the nuclei distorts the balance of spliced and unspliced mRNAs in a complex way. Their relative abundances can shift depending on the nuclear transport rates or the tendency to be present in the residual cytoplasmic structures remaining on the outer surface of the purified nuclei. While first applications of the existing RNA velocity model show promising results (Marsh & Blelloch, 2020), the assumptions such as constant degradation and nuclear export have not been conclusively verified, so it remains to be seen whether alternative models or normalization methods could provide consistent velocity estimates from single‐nucleus data.

### Batch effect removal

Current implementations are not designed to yield robust estimates across multiple samples with potential batch effects. While batch effect correction has been increasingly addressed in scRNA‐seq analysis, it is yet unclear how these methods can be extended to the non‐trivial setting with two connected modalities of unspliced and spliced abundances. When applying batch correction to each modality independently, it is likely that the relationship between the two modalities is not retained, which results in model misspecification. It becomes particularly limiting in the context of processes that must be sampled using time series designs, in which batch effects are introduced as cells are harvested at different time points. At the current state, we recommend fitting each sample separately, if potential batch effects cannot be ruled out. Coupled batch removal or state‐variable models are necessary to address this issue.

### Gene selection, visualization, and interpretation

The combination of velocities across genes is used to show the direction of movement of an individual cell in a dimensionality‐reduced embedding. Incorrect directions can not only result from erroneous velocity estimates, but also result from biases in the way velocities are projected.

For instance, only a selection of genes is used for projection as datasets are filtered to keep only genes that are informative of the variability in the data. In particular, intron proximity to the 3’ end may cause compositional bias affecting gene selection. Simultaneously, the interpretation of the projected velocities is hampered by the difficulty in identifying individual gene dynamics that give rise to the projections. For instance, projections can be distorted due to multiple dynamic processes that occur simultaneously in a specific regime, such as cell cycle and differentiation. Here, methods to assess gene selection bias, joint models for better latent space representations, and factor models to untangle compositional effects will be highly relevant.

## Conclusion

The data revolution in single‐cell biology, the detailed cellular maps of tissues, and the emergence of multi‐omics technologies provide unprecedented opportunities to analyze the complexity of biological systems. We have reviewed the current state of modeling kinetics in scRNA‐seq using RNA velocity and outlined conceptual and technical extensions that are necessary to account for recent and upcoming advances in single‐cell biology. With the ongoing endeavors of RNA velocity and its impact on various areas in cell biology, we envision that new directions in dynamic modeling will be enabled by this intriguing concept.

## Analyzed datasets

All scRNA‐seq datasets analyzed in this paper are published, publicly available, and directly accessible through https://scvelo.org, except for CD34^+^ cord blood cells. The hippocampal dentate gyrus neurogenesis datasets at P12 and P35 are available from the Gene Expression Omnibus repository (GEO) under accession number GSE95753, and the P0 and P5 hippocampus dataset under accession GSE104323. The mouse gastrulation atlas (Pijuan‐Sala *et al*, 2019) is available under accession number GSE87038. The human bone marrow data are available through the Human Cell Atlas data portal. The 68k PBMC data are available from the Short Read Archive under accession number SRP073767.

The results reported in this manuscript are available at https://scvelo.org/perspectives.

## Conflict of interest

VB is a full‐time employee of Cellarity Inc. and reports ownership interest in Cellarity Inc.; the present work was carried out at Helmholtz Munich. FJT reports receiving consulting fees from Roche Diagnostics GmbH and Cellarity Inc., and ownership interest in Cellarity Inc. and Dermagnostix. PVK serves on the Scientific Advisory Board to Celsius Therapeutics Inc. and Biomage Inc. The other author declares that they have no conflict of interest.
